# Focal Seizures Induced by Paraneoplastic Syndrome Leading to a Diagnosis of Small Cell Lung Carcinoma

**DOI:** 10.7759/cureus.62778

**Published:** 2024-06-20

**Authors:** Lisle W Blackbourn, Joseph Samaha, Manjari Uppu

**Affiliations:** 1 Neurology, University of Illinois College of Medicine Peoria, Peoria, USA; 2 Neurology, OSF Illinois Neurological Institute, Peoria, USA; 3 Medicine, Hotel-Dieu de France Hospital Faculty of Medicine, Saint Joseph University, Beirut, LBN

**Keywords:** small cell lung cancer, anti-hu, anna-1, seizure, anti-hu encephalitis, paraneoplastic encephalitis, paraneoplastic syndromes

## Abstract

Paraneoplastic neurological disorders are a rare complication of multiple neoplasms, such as lung, testis, and breast, and can be associated with positive antibody anti-Hu (anti-neuronal nuclear antibody type 1 or ANNA-1), anti-Ta, anti-Ma, and uncharacterized antibody, or be antibody-negative. Early treatment of the underlying tumor is the most likely modality that will lead to regression of the paraneoplastic neurological symptoms. Here, we present a case of a 73-year-old female with new-onset seizure activity from ANNA-1 encephalitis found to have undiagnosed small cell lung cancer to highlight the need for further workup for malignancy.

## Introduction

Paraneoplastic neurological disorders are a rare complication of multiple neoplasms, such as lung, testis, and breast, and can be associated with positive antibody anti-Hu (anti-neuronal nuclear antibody type 1 or ANNA-1), anti-Ta, anti-Ma, and uncharacterized antibody, or be antibody-negative [1]. They can vary from Lambert-Eaton myasthenic syndrome, sensory neuropathy, and autonomic dysfunction to limbic encephalitis and can be seen in up to 9% of small cell lung cancer (SCLC) diagnoses [2]. The diagnosis is made either by neuropathological examination or clinically if the patient develops compatible symptoms within four years of tumor diagnosis, after exclusion of other causes and with at least one of the following: cerebrospinal fluid (CSF) with inflammatory changes but negative cytology; magnetic resonance imaging (MRI) demonstrating temporal lobe abnormalities; and electroencephalography (EEG) showing epileptic activity in the temporal lobes [1,2]. CSF inflammatory changes can include pleocytosis, oligoclonal bands, increased immunoglobulins, or increased protein, while MRI can show abnormalities on T2-weighted images or atrophy seen on T1-weighted images in the temporal lobes [1]. Here, we present a case of a 73-year-old female with new-onset seizure activity and encephalopathy found to have SCLC.

## Case presentation

The patient was a 73-year-old female with a past medical history of intracranial aneurysm, hypertension, hyperlipidemia, rheumatoid arthritis of unknown seroreactivity, breast cancer treated with chemotherapy, and chronic obstructive pulmonary disease, who originally presented to the hospital with sepsis secondary to a *Staphylococcus* infection of unknown source. During a recent hospital admission a couple of months prior, the patient started to have new onset seizure-like activity of blank staring, lip smacking, and left facial twitching, for which she was initially started on levetiracetam 2000 milligrams (mg) twice per day (BID), and weaned to 750 mg in the morning and 1500 mg in the evening. Due to the patient's seizure history and concern for continued altered mental status, neurology was consulted.

On physical exam, the patient was oriented to person and place but not to the day of the week, month, or year. There were no cranial nerve deficits, and sensory, tone, reflexes, and coordination were all normal. The patient had 5/5 power in the bilateral upper extremities and 4+ out of 5 power in the bilateral lower extremities. However, over the course of her stay, the patient became gradually weaker in all muscle groups. The patient had no signs of autonomic dysfunction.

On EEG, the patient had intermittent left hemispheric delta theta slowing and rare focal epileptiform discharges in the left temporal region. While on EEG, the patient had multiple lip-smacking events concerning for seizure activity. There was no clear EEG correlation with these movements. Due to the patient's seizure history and altered mental status, the patient was loaded with 200 mg of lacosamide and started on 100 mg BID maintenance dosing after these events.

**Figure 1:**
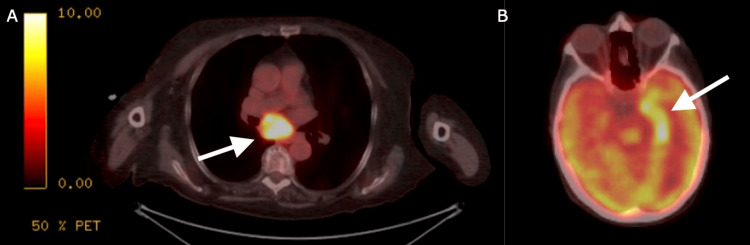
PET CT showing (A) a soft tissue mass in the lung suspicious for malignancy and (B) left hippocampal hypermetabolic activity suspicious for possible paraneoplastic encephalitis.

A non-contrast computed tomography (CT) of the head showed no acute abnormalities and a CT angiogram of the head and neck showed an incidental left anterior choroidal aneurysm. Due to the patient's unknown source of infection, the infectious disease had ordered a positron emission tomography CT (PET CT), which showed a soft tissue mass in the lung suspicious for malignancy and left hippocampal hypermetabolic activity suspicious for possible paraneoplastic encephalitis, as seen in Figure 1.

**Figure 2:**
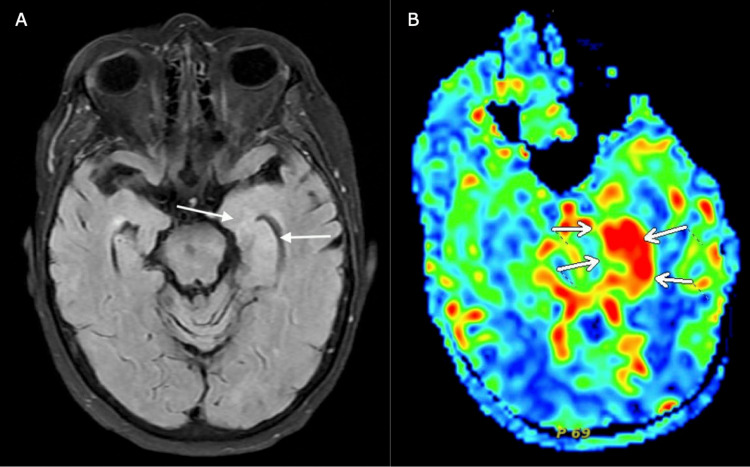
(A) MRI of the brain T2/fluid-attenuated inversion recovery (FLAIR) sequencing showing left mesial temporal lobe edema with contrast enhancement. (B) MRI of the brain showing increased cerebral blood flow in the same area.

An MRI of the brain with and without contrast showed persistent and worsening left mesial temporal lobe edema with ongoing contrast enhancement when compared to one done a couple of months prior when done for the patient's new onset seizure activity and increased cerebral blood flow in the same area, as seen in Figure 2.

A lumbar puncture was performed and indicated a noninfectious cause based on initial results. At this time, the patient was started on plasmapheresis for five days. At the end of treatment, the patient's weakness improved; however, her disorientation did not. Later the patient's CSF results started to come back. An encephalopathy/autoimmune evaluation panel (ENS2) through the Mayo Clinic had glutamic acid decarboxylase 65 (GAD65) antibody elevation and ANNA-1 reactivity. Reflex titer ANNA-1 testing resulted positive on two separate tests.

Fine-needle aspiration of a subcarinal lymph node was done, and overall, more than 95% of the tumor consisted of high-grade neuroendocrine carcinoma with features of small cell carcinoma. In addition, scattered small foci of very large neoplastic cells with eosinophilic cytoplasm were also noted. These clusters showed focal staining for P40, synaptophysin, and cytokeratin 20 (Ck20).

The patient was diagnosed with SCLC and suspected encephalitis was believed secondary to paraneoplastic syndrome. The patient’s levetiracetam was switched to valproate 500 mg BID due to issues with anxiety prior to discharge. The patient was started on chemotherapy for the SCLC; however, she eventually passed away.

## Discussion

It was determined that our patient had paraneoplastic encephalitis secondary to ANNA-1 due to her clinical presentation, MRI findings, and antibody testing. GAD65 was deemed not clinically significant as ANNA-1 is much more likely to cause paraneoplastic neurological disorders [2]. Further, GAD65 is not very specific and can be positive in a wide variety of autoimmune disorders.

Although a PET CT scan in our patient showed a soft tissue mass in the lung suspicious for malignancy prior to the results of ANNA-1 encephalitis testing coming back, many times the diagnosis of ANNA-1 encephalitis comes before any known mass is found. ANNA-1 encephalitis is estimated to precede the cancer diagnosis in 60-89% of cases and is most frequently associated with SCLC (94%) [1,3]. No cancer is found in approximately 15% of cases [4,5]. Therefore, it is crucial for an early, thorough, and extensive cancer screening via whole-body CT scan or PET scan when a diagnosis of ANNA-1 encephalitis is made. One case even reported ANNA-1 limbic encephalitis nine years before the appearance of a tumor [6].

Localization and clinical manifestations

ANNA-1 paraneoplastic neurological disorders can be divided into limbic encephalitis, peripheral neuropathy, cerebellar ataxia, and multifocal neurological involvement [3,4].

Symptoms can vary from sensory neuronopathy, memory deficits, seizures, personality, and behavioral changes, to bulbar, medullary, or pontine symptoms (dysphagia, dysarthria, central hypoventilation, cranial nerve palsy, gait ataxia) [1,7].

Paraclinical findings

As discussed previously, paraclinical findings are essential for the diagnosis of ANNA-1 associated-neurological disorders. In fact, brain MRI and EEG typically show limbic involvement and temporal lobes abnormalities, and up to half of patients could have extratemporal slowing, epileptiform discharges, or epilepsia partialis continua on the EEG [1,8].

CSF analysis could show pleocytosis, elevated proteins, oligoclonal bands, and/or intrathecal IgG with negative cytology [1]. In addition, a positive ANNA-1 (anti-Hu antibody) in the serum and/or in the CSF is a useful diagnostic marker and can be found in around 36% of patients with paraneoplastic limbic encephalitis. ANNA-1 antibody testing has been shown to be extremely specific at 99% and have a good sensitivity of 82%; however, this was studied in patients with paraneoplastic sensory neuropathy [9].

Treatment and prognosis

Early treatment of the underlying tumor is the most likely modality that will lead to the regression of the paraneoplastic neurological symptoms [1,4]. Some patients could benefit from immunosuppressive therapies like immunoglobulin, corticosteroids, plasma exchange, and rituximab or human chorionic gonadotropin [4,10,11].

In a cohort of 200 patients with ANNA-1 paraneoplastic encephalomyelitis, the median survival was 11.8 months, with a three-year survival of 20% and neurological complications being the most common cause of death [4]. This is significantly less than the survival time of 43 months from a cohort of 63 patients who had paraneoplastic neurological disorders and in line with a cohort of 24 patients who had a survival time of 11.5 months [2,12].

## Conclusions

Here, we present a case of new-onset seizure activity stemming from ANNA-1 encephalitis to highlight the need for further workup for malignancy. ANNA-1 is associated with SCLC and can precede the cancer diagnosis in a significant proportion of patients. Therefore, it is crucial for an early, thorough, and extensive screening via whole-body CT scan or whole-body PET scan. Early treatment of the underlying tumor is the most likely modality that will lead to regression of the paraneoplastic neurological symptoms. Untreated neurological complications, such as seizure activity, from the ANNA-1 encephalitis can lead to death, making treatment of the underlying tumor and therefore initial diagnosis of malignancy important.
